# P2X7 promotes metastatic spreading and triggers release of miRNA-containing exosomes and microvesicles from melanoma cells

**DOI:** 10.1038/s41419-021-04378-0

**Published:** 2021-11-16

**Authors:** Anna Pegoraro, Elena De Marchi, Manuela Ferracin, Elisa Orioli, Michele Zanoni, Cristian Bassi, Anna Tesei, Marina Capece, Emi Dika, Massimo Negrini, Francesco Di Virgilio, Elena Adinolfi

**Affiliations:** 1 Department of Medical Sciences, Section of Experimental Medicine, University of Ferrara, Ferrara, Italy; 2 Department of Experimental, Diagnostic and Specialty Medicine (DIMES), University of Bologna, Bologna, Italy; 3 IRCCS Istituto Romagnolo per lo Studio dei Tumori (IRST) “Dino Amadori”, Meldola, Italy; 4 Department of Translational Medicine and for Romagna, and “Laboratorio per le Tecnologie delle Terapie Avanzate” (LTTA), University of Ferrara, Ferrara, Italy; 5 Department of Cancer Biology and Genetics, College of Medicine, The Ohio State University and James Comprehensive Cancer Center, Columbus, OH 43210 USA; 6 Dermatology Unit, IRCCS Azienda Ospedaliero-Universitaria di Bologna, Bologna, Italy

**Keywords:** Ligand-gated ion channels, Metastasis, Melanoma, Ion channel signalling, miRNAs

## Abstract

Tumor growth and metastatic spreading are heavily affected by the P2X7 receptor as well as microvesicles and exosomes release into the tumor microenvironment. P2X7 receptor stimulation is known to trigger vesicular release from immune and central nervous system cells. However, P2X7 role in microvesicles and exosomes delivery from tumor cells was never analyzed in depth. Here we show that P2X7 is overexpressed in patients affected by metastatic malignant melanoma and that its expression closely correlates with reduced overall survival. Antagonism of melanoma cell-expressed P2X7 receptor inhibited in vitro anchorage-independent growth and migration and in vivo dissemination and lung metastasis formation. P2X7 stimulation triggered the release of miRNA-containing microvesicles and exosomes from melanoma cells, profoundly altering the nature of their miRNA content, as well as their dimensions and quantity. Among the more than 200 miRNAs that we found up-or-down-modulated for each vesicular fraction tested, we identified three miRNAs, miR-495-3p, miR-376c-3p, and miR-6730-3p, that were enriched in both the exosome and microvesicle fraction in a P2X7-dependent fashion. Interestingly, upon transfection, these miRNAs promoted melanoma cell growth or migration, and their vesicular release was minimized by P2X7 antagonism. Our data unveil an exosome/microvesicle and miRNA-dependent mechanism for the pro-metastatic activity of the P2X7 receptor and highlight this receptor as a suitable prognostic biomarker and therapeutic target in malignant melanoma.

## Introduction

The P2X7 receptor (P2X7R) is an extracellular ATP (eATP)-gated cation channel [1]. P2X7R expression or stimulation activates the PI3K/AKT and VEGF signaling pathways [2–4], enhances glycolytic and oxidative metabolism [5], promotes motility [6], causes the release of cathepsins [7], promotes transendothelial migration [8], and increases in vivo tumor growth [2]. Furthermore, the P2X7R affects the eATP concentration of the tumor microenvironment [9, 10]. Depending on the activation level and the specific immune cell type involved, the P2X7R acts as a crucial determinant of tumor–host interaction by driving pro-inflammatory or tolerogenic responses [9, 11, 12]. Thirteen splicing variants of the human P2X7R have been identified, including P2X7B, which is involved in tumor growth and resistance to chemotherapy [13, 14].

Melanoma-bearing mice were among the first models in which P2X7R blockers showed an efficacious reduction of primary cancer [2, 15]. Nevertheless, studies relating P2X7 expression with melanoma overall survival and metastasis in patients cohorts, as well as preclinical data supporting the efficacy of P2X7 targeting in reducing melanoma spreading, were missing. Herein we investigate the role of P2X7R in melanoma progression and dissemination, focusing on the release of microvesicles (MV) and exosomes (EXO) and their miRNA content.

Extracellular vesicles (EV), including MV and EXO, are released by all cell types, including cancer, neuronal and immune cells, and transfer cellular components from donor to target cells in the vicinity or outlying districts [16]. MV and EXO are known mediators of pro-metastatic activities, including melanoma migration, local invasion, preconditioning, and colonization of secondary sites [17, 18]. These activities depend on EVs content, including proteins, lipids, and nucleic acids, among which miRNAs [19]. miRNAs are small non-coding RNA involved in post-transcriptional gene regulation by binding to target mRNAs in a sequence-dependent way and inhibiting its translation. Therefore, miRNAs can affect the expression of both oncogenes and oncosuppressors, thus promoting carcinogenesis and metastatic dissemination [20]. Regardless of their established role in cancer, mechanisms underlying EV release and modulation of metastasis-promoting activity remain elusive [21]. P2X7R activation is known to cause MV release from cells of the monocyte/macrophage lineage and astrocytes [22–26], but its role in their delivery from tumor cells was never investigated in depth. Here we show that the P2X7R is up-modulated in poor prognosis melanoma patients, and its blockade reduces metastatic spreading in multiple in vitro and in vivo melanoma models. Moreover, P2X7R stimulation emerges as a powerful stimulus for MV and EXO-mediated miRNA release.

## Results

### P2X7R expression associates with reduced overall survival and metastasis in melanoma patients

**Fig. 1:**
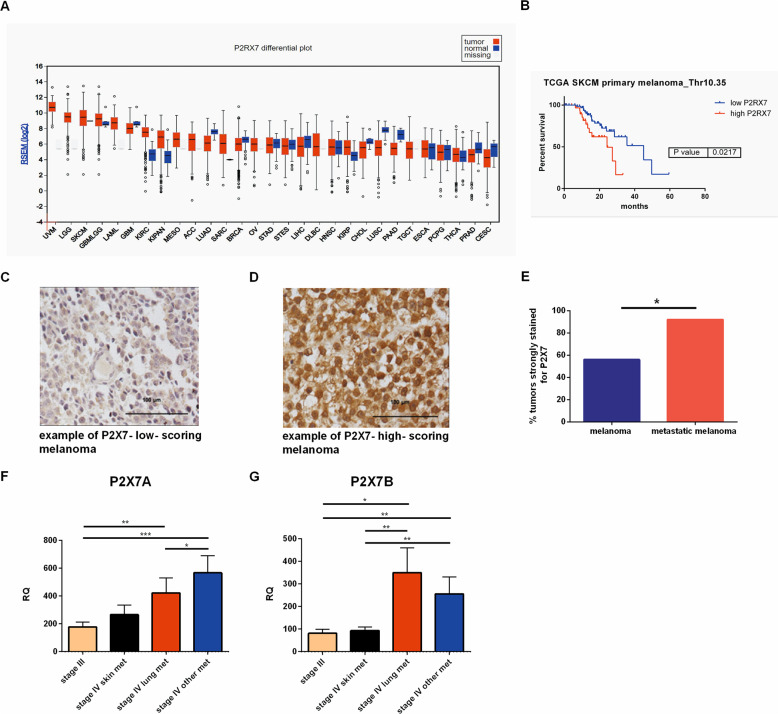
P2X7R over-expression associates with reduced overall survival and metastasis in melanoma patients. **A** expression of P2X7R in tumor and normal samples from TCGA database. Normalized expression of P2X7R, detected using RNA sequencing, was obtained from Firebrowse database. Tumor types were ordered according to log2 P2X7R expression. **B** overall survival of melanoma patients with low or high P2X7R expression (threshold 10,35). **C** representative image of weak immunohistochemical staining for P2X7R of a human melanoma sample. **D** representative image of strong immunohistochemical staining for P2X7R of a human metastatic melanoma specimen. **E** Percentage of metastatic (red, *N* = 14) and non-metastatic melanoma (blue, *N* = 25) samples with strong positivity for P2X7R *p* = 0.0283 exact Fishers’ test. **F** and **G** mRNA level of P2X7RA and P2X7RB evaluated in 77 cDNAs from melanoma patients subdivided in stage III (*N* = 27), stage IV with skin metastasis (*N* = 15), stage IV with lung metastasis (*N* = 10), and stage IV with metastasis in other distant organs (*N* = 14). *p < 0.05, **p < 0.01, ***p < 0.001 by Student’s *t*-test.

The role of the P2X7R has been widely investigated in experimental melanoma models, but little is known about its expression and prognostic potential in patients’ cohorts [27]. The P2X7R is highly expressed in both cutaneous (SKCM) and uveal (UVM) melanomas from the TCGA (Fig. 1A). Moreover, in the SKCM cohort, we found a significant association between P2X7R overexpression and reduced overall survival (Fig. 1B). To further strengthen these data, we performed immunohistochemical analysis of P2X7R in 39 human melanoma specimens, including metastatic cases. Almost all samples tested stained positive for P2X7R. However, 92% of metastatic patients were strongly positive compared to the 56% of primary cases (Fig. 1C–E). We extended our analysis to the expression of P2X7R splicing variants A and B, which were associated with malignant transformation [13, 14, 28, 29]. In an array of 77 cDNA from melanoma specimens, we observed that both isoforms significantly increased in samples from stage IV melanomas with metastasis in the lungs and other organs distal from the primary tumor site, such as the liver and brain. In comparison, samples from stage III and IV with metastases localized to the skin showed lower expression of both P2X7A and B (Fig. 1F, G).

### P2X7R antagonists reduce Sk-Mel-28 and Ma-Mel-19 melanoma cells proliferation, anchorage-independent growth, and migration

**Fig. 2:**
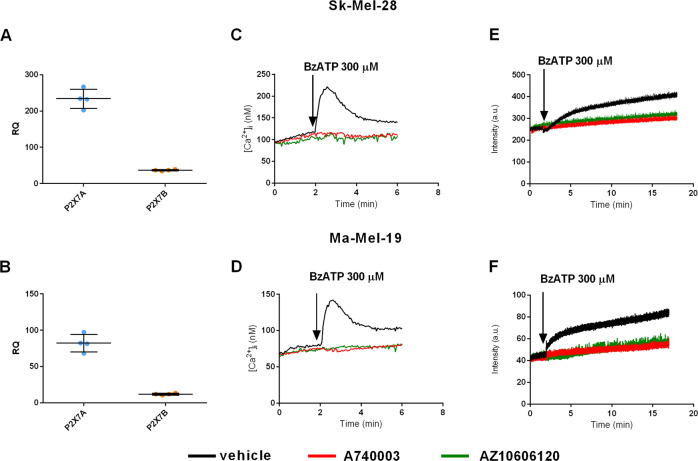
Sk-Mel-28 and Ma-Mel-19 human melanoma cell lines express a functional P2X7 receptor. **A** mRNA expression of P2X7A and P2X7B in Sk-Mel-28 cells evaluated by real time-PCR (*N* = 4). **B** mRNA expression of P2X7A and P2X7B in Ma-Mel-19 cells evaluated by real-time PCR (*N* = 4). P2X7 receptor activity as a channel was evaluated in **C** Sk-Mel-28 cells and **D** Ma-Mel-19 cells by measuring variations in [Ca^2+^]_I_ following stimulation with P2X7R agonist BzATP (300 μM). The P2X7 receptor antagonists A740003 (20 μM) and AZ10606120 (2 μM) completely obliterated the effect of BzATP. P2X7 activity as a large solute (up to 900 Da) pore was evaluated in **E** Sk-Mel-28 cells and **F** Ma-Mel-19 cells by measuring ethidium uptake following stimulation with BzATP (300 μM). The P2X7 receptor antagonists A740003 (20 μM) and AZ10606120 (2 μM) blocked the effect of BzATP.

**Fig. 3:**
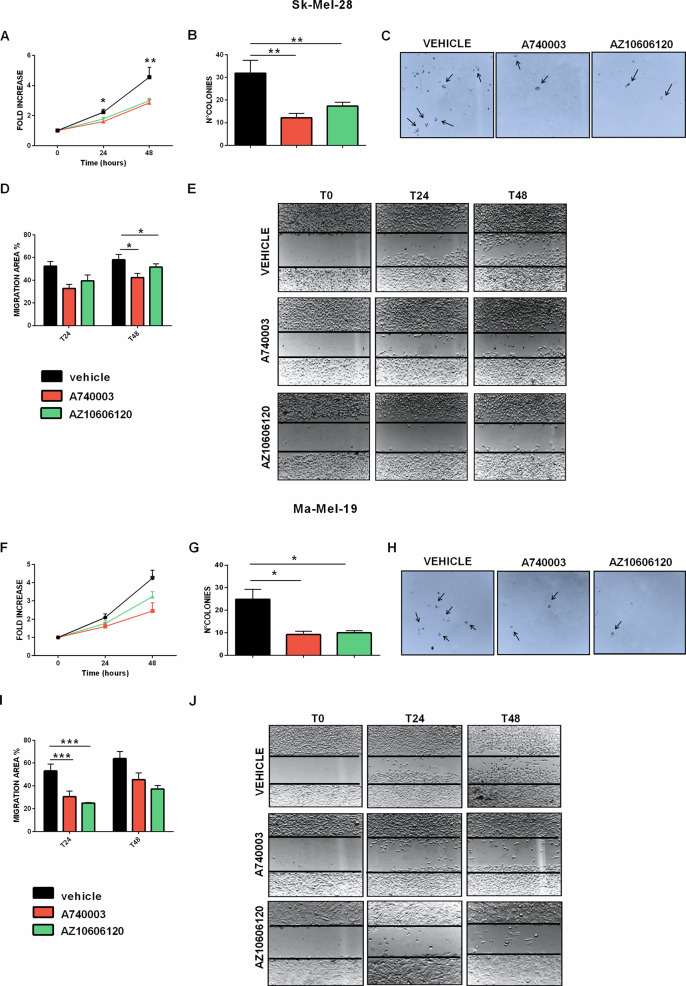
P2X7 antagonists reduce melanoma cell proliferation, anchorage-independent growth, and migration. **A** Sk-Mel-28 cells were treated with P2X7 antagonists A740003 (20 μM) and AZ10606120 (2 μM) and counted at time 0 and after 24 and 48 h of incubation. Data are presented as a fold increase of time 0 (*N* = 10, five independent experiments). **B** number of colonies formed by Sk-Mel-28 cells in soft agar plates after 20 days of incubation with a vehicle, A740003 (20 μM) or AZ10606120 (2 μM) (*N* = 6, three independent experiments). **C** representative pictures showing colonies formed by Sk-Mel-28 cells. **D** quantification of the migratory ability of Sk-Mel-28 cells expressed as the percentage of the migration area evaluated by scratch closure assay at 24 and 48 h of incubation with vehicle, A740003 (20 μM) or AZ10606120 (2 μM) (*N* = 6, three independent experiments). **E** representative images of Sk-Mel-28 cells scratches taken at times 0, 24 and 48. **F** Ma-Mel-19 cells were treated with P2X7 antagonists A740003 (20 μM) and AZ10606120 (2 μM) and counted at time 0 and after 24 and 48 h of incubation. Data are presented as a fold increase of time 0 (*N* = 7, in three independent experiments). **G** number of colonies formed by Ma-Mel-19 cells in soft agar plates after 20 days of incubation with vehicle, A740003 (20 μM) and AZ10606120 (2 μM) (*N* = 6, three independent experiments). **H** representative pictures showing colonies formed by Ma-Mel-19 cell. **I** quantification of the migratory ability of Ma-Mel-19 cells expressed as the percentage of the migration area evaluated by scratch closure assay at 24 and 48 h of incubation with vehicle, A740003 (20 μM) and AZ10606120 (2 μM) (*N* = 6, three independent experiments). **J** representative images of Ma-Mel-19 cells scratches taken at times 0, 24, and 48. Data are shown as the mean ± SEM, *p < 0.05, **p < 0.01, ***p < 0.001, Student’s *t*-test.

Figure 2 shows P2X7A and B isoform expression, BzATP-induced intracellular Ca^2+^ changes, and ethidium bromide uptake in Sk-Mel-28 and Ma-Mel-19 human melanoma cells (Fig. 2A–F). These responses were fully inhibited by pretreatment with two unrelated P2X7R selective blockers, i.e., A740003 and AZ10606120. These cell lines were used in the following experiments to investigate the effects of P2X7R antagonists on proliferation, malignant transformation, and migration. The P2X7R blockers reduced the growth of both Sk-Mel-28 and Ma-Mel-19 cells (Fig. 3A, F), suppressed anchorage-independent growth in the soft agar assay (Fig. 3B, C, G, H), and inhibited migratory ability in the scratch recovery assay (Fig. 3D, E, I, J).

### P2X7R blockade inhibits metastatic melanoma spreading in murine experimental models

**Fig. 4:**
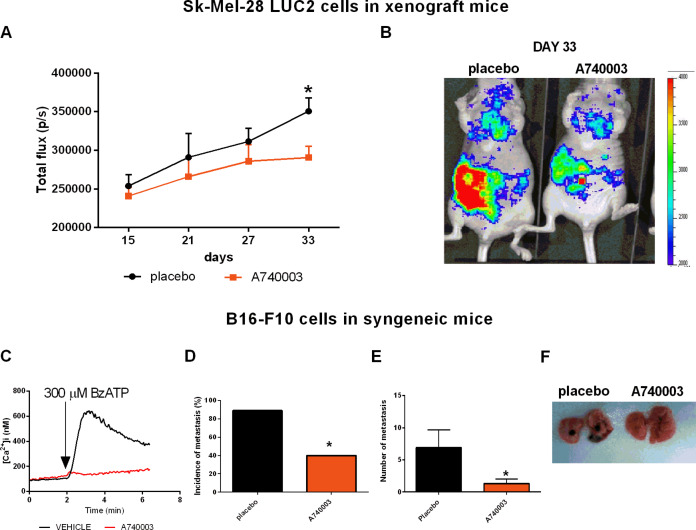
P2X7 antagonist A740003 reduces in vivo melanoma cell dissemination and metastasis formation. **A** Photons emission measured every 6 days in mice in which Sk-Mel-28 cells expressing the luciferase Luc2 were injected into the tail vein and treated with a placebo (PBS, 0.005% DMSO) or A740003 (50 μg/kg) every 3 days, starting from the day of inoculum of tumor cells. Luminescence emission is presented as total flux (p/s). Data are shown as the mean ± SEM, *N* = 8 per condition. *p < 0.05, Student’s *t*-test. **B** Representative image of Luc2 luminescence emission in placebo and A740003 treated mice at day 33. **C** B16-F10 cells show an increase in [Ca^2+^]_I_ upon P2X7R stimulation with Bz-ATP that is reversed by treatment with 20 µM A740003. **D**–**F** B16-F10 cells were injected into the tail vein of C57/bl6 mice, which were i.p. administered with A740003 (50 μg/kg) or placebo (PBS, 0.005% DMSO) every 3 days starting from the day of inoculum of cancer cells. *N* = 8 per condition. **D** Percentage of mice treated with placebo or A740003 that developed at list one metastasis. *p = 0.0114, Fisher Exact’s Test. **E** Number of metastasis growth in the lungs of placebo or A740003 treated mice. Data are shown as the mean ± SEM. *p < 0.05, Student’s *t*-test. **F** Representative images of lungs of mice treated with placebo or A740003.

In vitro data reported in Fig. 3 strongly suggest a role for P2X7R in melanoma spreading. To verify the P2X7R metastases-promoting capacity, we evaluated the effect of P2X7R blockade with A740003, which was previously shown to in vivo reduce primary melanoma growth and activate antitumoral immune responses [9], in murine models of metastasis. To this aim, we stably transfected Sk-Mel-28 cells with an intracellular luciferase, thus enabling us to monitor in vivo metastatic dissemination with a small animal total body luminometer. P2X7R antagonism significantly slowed down melanoma spreading, reducing tumor dissemination by almost 50% at post inoculum day 33 (Fig. 4A, B). Xenotransplantation is limited by the lack of a fully active host immune system. Therefore, we performed an in vivo dissemination assay in a syngeneic murine model using P2X7R-expressing B16-F10 melanoma cells (Fig. 4C). In this model at post-inoculum day 18, 90% of the control mice showed visible lung metastases against 40% of the mice treated with P2X7R antagonist A740003 (Fig. 4D). Moreover, also the number of lung metastases was significantly reduced by P2X7R blockade (Fig. 4E, F).

### P2X7R triggers EV release from melanoma cells

**Fig. 5:**
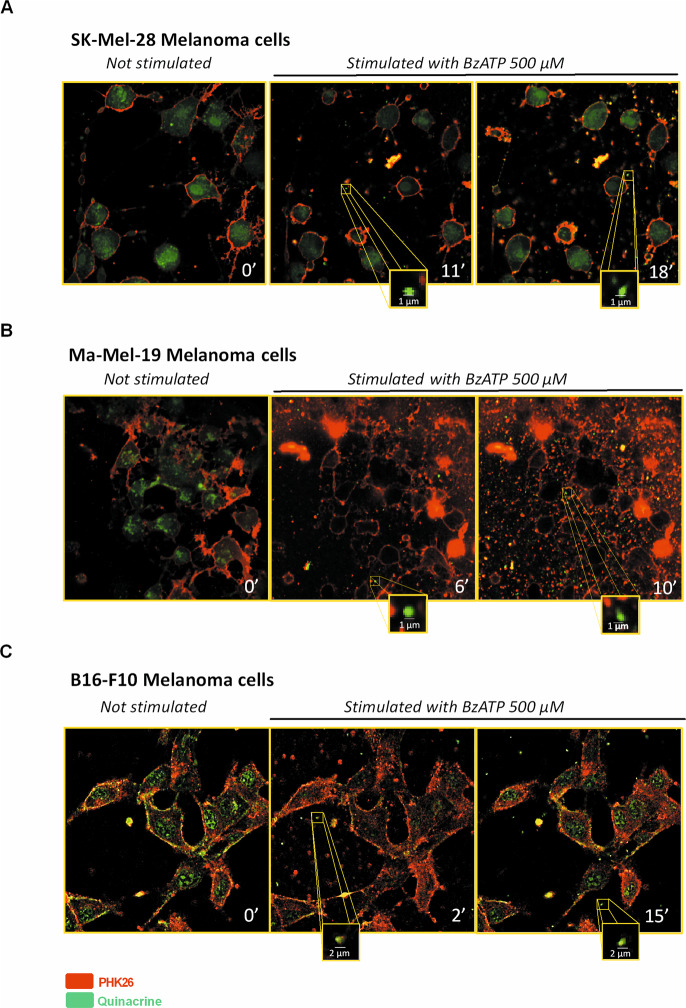
Human and murine melanoma cells release vesicles upon stimulation of P2X7R. Representative images acquired with a confocal microscope of **A** Sk-Mel-28 cells, **B** Ma-Mel-19 cells, and **C** B16-F10 cells before and after stimulation with the P2X7 agonist BzATP (500 μM). The plasma membrane was stained with the red dye PKH26GL, while nucleic acid content was labeled with quinacrine.

**Fig. 6:**
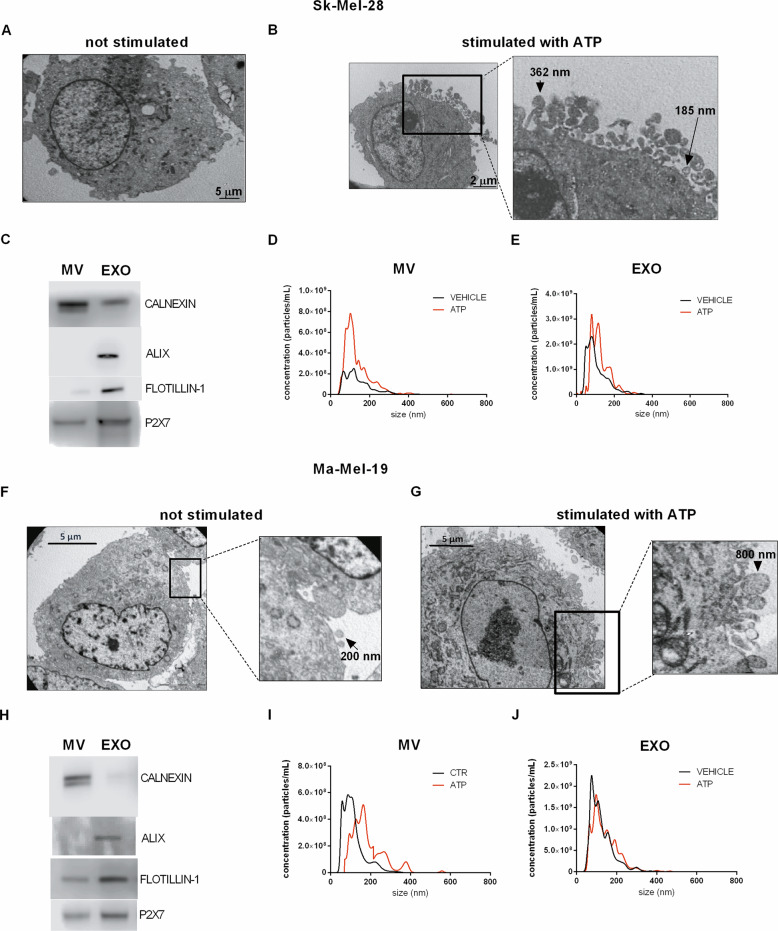
Characterization of vesicles released from Sk-Mel-28 and Ma-Mel-19 cells. **A** Representative electron microscopy image of an unstimulated Sk-Mel-28 cell (magnification × 4000). **B** Representative electron microscopy image of Sk-Mel-28 cell stimulated with 3 mM ATP (magnification × 8000). **C** Western blot analysis of calnexin (95 kDa), Alix (95 kDa), flotillin-1 (49 kDa), P2X7 (75 kDa) in MV, and EXO isolated from Sk-Mel-28 cells treated with 3 mMATP. **D** Representative graphs of size distribution and concentration of MV released from Sk-Mel-28 cells stimulated with a vehicle or ATP. **E** Representative graphs of size distribution and concentration of EXO released from Sk-Mel-28 cells stimulated with a vehicle or ATP. **F** Electron microscopy image of an unstimulated Ma-Mel-19 cell (magnification × 4000). **G** Electron microscopy image of a Ma-Mel-19 cell stimulated with 3 mM ATP (magnification × 4000). **H** Western blot analysis of calnexin (95 kDa), Alix (95 kDa), flotillin-1 (49 kDa), P2X7 (75 kDa) in MV, and EXO isolated from Ma-Mel-19 cells treated with 3 m MATP. **I** Representative graphs of size distribution and concentration of MV released from Ma-Mel-19 cells stimulated with a vehicle or ATP. **J** Representative graphs of size distribution and concentration of EXO released from Ma-Mel-19 cells stimulated with a vehicle or ATP. Nanosight Analysis of five different videos allowed to obtain data relative to vesicle concentration and size.

Figure 5 shows vesicular release from Sk-Mel-28 (Fig. 5A and Movie 1), Ma-Mel-19 (Fig. 5B and Movie 2), and B16-F10 (Fig. 5C and Movie 3) melanoma cells triggered by P2X7R agonist Bz-ATP. Interestingly, these vesicles also stained positively for quinacrine (in green), thus suggesting a possible content of nucleic acids and ATP (Fig. 5A–C). Vesicles released from Sk-Mel-28 and Ma-Mel-19 were further characterized by electron microscopy, Western blot for MV and EXO markers, and nanoparticle tracking analysis (Fig. 6). Electron microscopy analysis of cells treated or not with ATP allowed us to estimate the presence of EV on the cell surface in both cases and suggested an increase in their number following P2X7 stimulation (Fig. 6A, B, F, G). The heterogeneous size of the particles (100–800 nm) led us to hypothesize P2X7-dependent release of different types of EV from melanoma cells. Vesicular fractions isolated by ultracentrifugation from both cell lines were confirmed to include both MV and EXO by immunostaining with Calnexin (MV marker), Alix, and Flottilin (EXO markers) (Fig. 6C, H and Supplementary Fig. 1). Nanoparticle-tracking analysis allowed us to estimate an ATP-dependent increase in the number of MV released by Sk-Mel-28 and in the size of EV released by Ma-Mel-19 (Fig. 6D, E, I, J and Supplementary Fig. 2).

### ATP stimulation modifies the miRNA content of MV and EXO

**Fig. 7:**
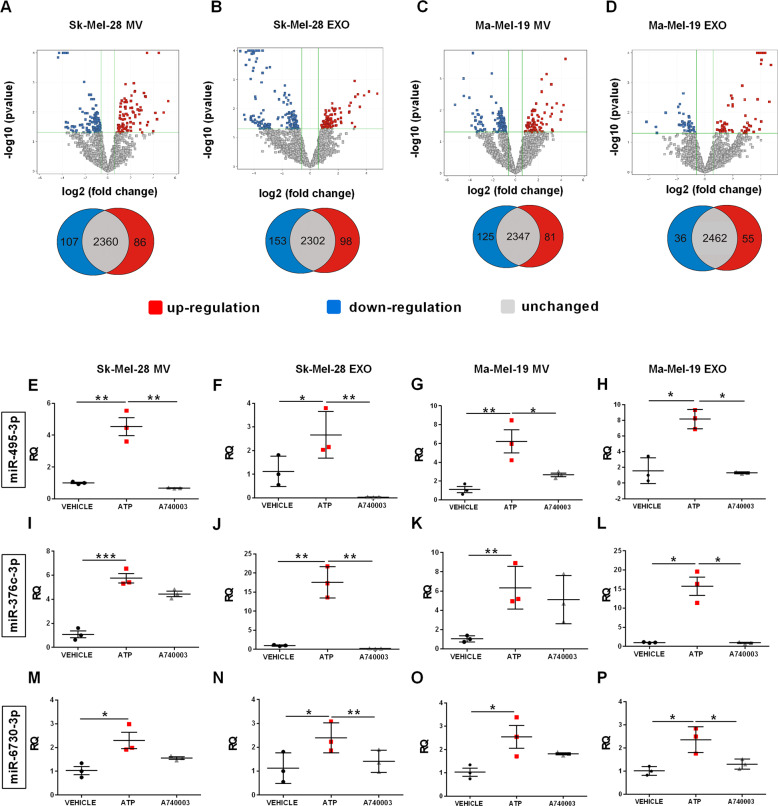
ATP stimulation alters miRNAs content of EV released from melanoma cells. **A–D** Volcano plot, and Venn diagram of differentially expressed miRNAs in MV and EXO vesicle fractions released from Sk-Mel-28 (**A**, **B**) and Ma-Mel-19 (**C**, **D**) cells following stimulation with 3 mM ATP (*N* = 3, three independent experiments). **E**–**P** Validation of selected miRNA expression levels by RT-PCR in MV and EXO from Sk-Mel-28 and Ma-Mel-19 cells following stimulation with 3 mM ATP. When required, 20 μM A740003 was administered together with ATP. Expression levels of hsa-miR-495-3p in **E** Sk-Mel-28 MV, **F** Sk-Mel-28 EXO, **G** Ma-Mel-19 MV, **H** Ma-Mel-19 EXO. Expression levels of hsa-miR-376c-3p in **I** Sk-Mel-28 MV, **J** Sk-Mel-28 EXO, **K** Ma-Mel-19 MV, **L** Ma-Mel-19 EXO. Expression levels of hsa-miR-6730-3p in **M** Sk-Mel-28 MV, **N** Sk-Mel-28 EXO, **O** Ma-Mel-19 MV, **P** Ma-Mel-19 EXO. Expression levels of the miRNAs are normalized on RNU6-1. *N* = 3, three independent experiments. *p < 0.05, **p < 0.01, ***p < 0.001 by Student’s *t*-test.

**Fig. 8:**
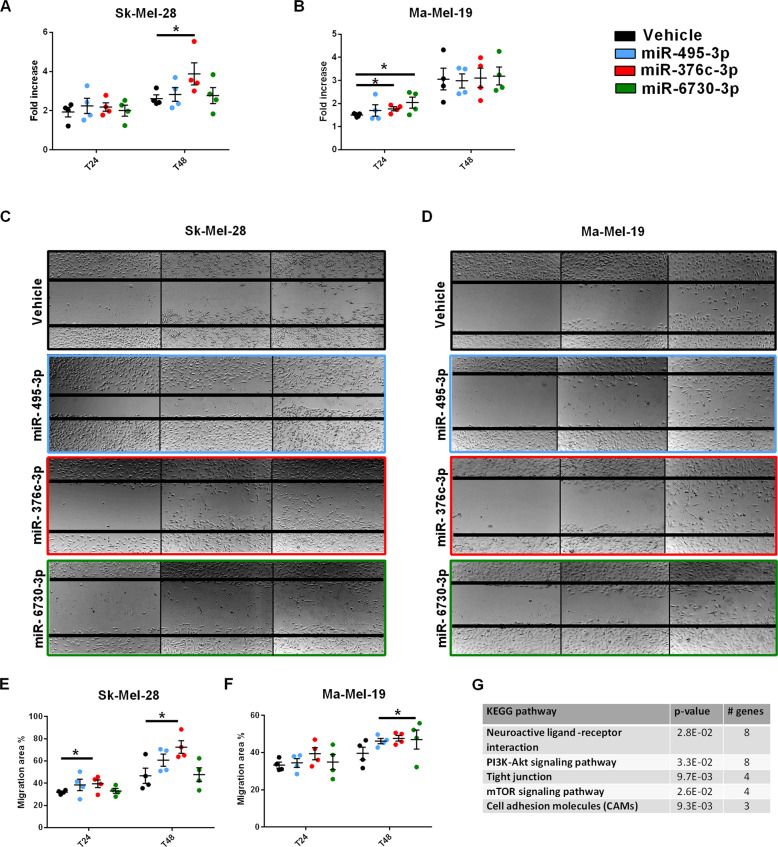
Effect of miR-495-3p, miR-376c-3p, and miR-6730-3p transfection on proliferation and migration of Sk-Mel-28 and Ma-Mel-19 cells. **A** Sk-Mel-28 or **B** Ma-Mel-19 cells were transfected with miRNA mimics for miR-495-3p, miR-376c-3p, miR-6730-3p and scrambled control. Cells were counted for 3 days, starting 24 h after transfection. Data are presented as a fold increase on time 0. **C**–**F** Sk-Mel-28 and Ma-Mel-19 cells were transfected with the above-described miRNA mimics or control. The scratch recovery assay was performed starting 24 h after transfection in serum-free medium **C** Representative images of scratches taken at time 0, 24 and 48 h in Sk-Mel-28. **D** Representative images of scratches taken at time 0, 24, and 48 h in Ma-Mel-19. **E** Quantification of the migratory ability of Sk-Mel-28 cells expressed as the percentage of the migration area evaluated after 24 and 48 h from the scratch formation. **F** Quantification of the migratory ability of Ma-Mel-19 cells expressed as the percentage of the migration area assessed after 24 and 48 h from the scratch formation. **G** Biological pathways regulated by all three tested miRNAs. Data are shown as the mean ± SEM of four independent experiments (*N* = 4). *p < 0.05 by Student’s *t*-test.

Analysis by small RNA sequencing of the miRNA content of MV and EXO released from Sk-Mel-28 and Ma-Mel-19 cells, before and after ATP stimulation, shows a different global miRNA expression profile (Fig. 7A–D). On a total of 2553 miRNAs analyzed, P2X7R stimulation caused differential expression of 251 miRNAs in Sk-Mel-28 EXO (98 up- and 153 down-regulated), of 193 miRNAs in Sk-Mel-28 MV (86 up- and 107 down-regulated), of 91 miRNAs in Ma-Mel-19 EXO (55 up- and 36-down regulated), and of 206 miRNAs in Ma-Mel-19 MV (81 up- and 125 down-regulated) (Fig. 7A–D). Thus, these data show a profound P2X7R-dependent alteration of EV miRNA content. To confirm these data and investigate the pathophysiological meaning, we selected three miRNAs showing the higher overexpression in MV and EXO released from both Sk-Mel-28 and Ma-Mel-19 upon P2X7R stimulation: miR-495-3p, miR-6730-3p, and miR-376c-3p. RT-PCR confirmed data from NGS analysis for all three miRNAs (Fig. 7E–P). Interestingly, P2X7R blockade with A740003 reduced the EV content of these three miRNAs. We investigated miR-495-3p, miR-376c-3p, and miR-6730-3p effects on cell proliferation and migration following their transfection into Sk-Mel-28 and Ma-Mel-19 (Fig. 8). Two miRNAs (miR-376c-3p and miR-6730-3p) increased melanoma cell growth (Fig. 8A, B), and miR-376c-3p also increased migration of both Sk-Mel-28 (Fig. 8C, E) and Ma-Mel-19 (Fig. 8D, F) in the scratch closure assay. These data suggest P2X7R stimulation causes the release of MV and EXO-containing miRNAs endowed with the ability to support prometastatic activity. Interestingly, analysis of intracellular pathways potentially targeted by miR-495-3p, miR-376c-3p, and miR-6730-3p revealed several signaling cascades related to P2X7R activity and cancer, PI3K/Akt and mTOR included, and to migration and intravasation (Fig. 8G).

## Discussion

Cutaneous melanoma is the most lethal skin cancer, responsible for more than 50,000 cancer-related deaths per year worldwide. While early-stage melanomas can be effectively treated with surgery, metastatic forms are often resistant to anti-cancer therapies [30]. Therefore, identifying novel prognostic markers and therapeutic targets for the metastatic forms of the disease is eagerly needed. An oncogenic role of P2X7R in murine models of primary melanoma was previously reported [2, 9, 15]. In the present study, we extend these findings by demonstrating by in silico analysis that the P2X7R is overexpressed in uveal and cutaneous melanomas and associates with reduced overall survival in the skin forms of the disease. TCGA analysis also confirmed upregulation of the P2X7R in other tumors previously shown to overexpress the receptor, such as acute myeloid leukemia [14] and glioma [31]. Moreover, we demonstrated by immunohistochemistry and real-time PCR an increase in P2X7R expression in metastatic forms of melanoma.

Interestingly, both P2X7A and P2X7B splice variant expression was almost doubled in metastatic forms disseminated to the lungs, brain, and liver. These data suggesting the involvement of both P2X7R isoforms in metastatic melanoma were confirmed by following experiments. P2X7R antagonists, equally targeting the two splice variants [13], significantly reduced proliferation of the metastatic melanoma cell lines Sk-Mel-28 and Ma-Mel-19 and restricted their ability to grow in and infiltrate a soft agar matrix. P2X7R blockers also inhibited melanoma cell migration in the scratch assay. These data were further confirmed in murine models of melanoma dissemination obtained, respectively, by i.v. injection Sk-Mel-28 cells into *nude/nude* mice or B16-F10 murine melanoma cells into an immune-competent host. P2X7R antagonism significantly reduced the dissemination of Sk-Mel-28 cells and nearly abrogated lung metastasis of B16-F10 cells. To our knowledge, this is the first demonstration that P2X7R blockade is effective in reducing melanoma metastasis in vivo, confirming data obtained in other cancer models [6, 32, 33]. Data obtained in the B16-F10 model that does not express the P2X7RB isoform suggests that P2X7RA blockade will be sufficient to reduce melanoma spreading. However, it does not exclude a role for isoform B in human melanoma dissemination as P2X7RB is expressed in Sk-Mel-28 cells and targeted by A740003 [13].

Increasing evidence suggests that the release of MV and EXO from tumor cells promotes metastasis formation [17, 18]. The P2X7R was previously shown to trigger vesicle release from immune cells and astrocytes [22, 34], but evidence that it might also trigger EV discharge from tumor cells was anecdotical [35]. Herein, we demonstrate that melanoma cells release EV when stimulated with the P2X7R agonist Bz-ATP. Interestingly, these vesicles were also positive for quinacrine staining, thus suggesting that they might contain nucleic acids, ATP included. Electron microscopy analysis suggested P2X7R-dependent release of both MV and EXO as the EV size spanned from 100 to 1000 nm. The presence of both vesicular sub-populations was confirmed by high-speed centrifuge fractionation and immunoblotting with specific MV and EXO markers. The nano-sight technique confirmed EV dimensions and demonstrated that P2X7R stimulation increases both the number and size of vesicles. P2X7R activation strikingly changed the miRNA content of MV and EXO released by both SK-Mel-28 and Ma-Mel-19 melanoma cells causing the up or down-regulation of more than 200 miRNAs for each vesicle population tested. Such a profound change in the miRNA composition is very likely to radically affect the transforming and migratory properties of melanoma cells favoring metastases engraftment. Incidentally, P2X7R-dependent stimulation of release of miRNA-containing EV is highly probable in the eATP-rich melanoma tumor microenvironment [9, 36]. Three miRNAs (miR-495-3p, miR-376c-3p, and miR-6730-3p) were strongly upregulated in all vesicles subtypes. To evaluate their role in tumor proliferation and metastasis, we transfected these miRNA into melanoma cells.

Interestingly, two miRNAs (miR-376c-3p and miR-6730-3p) increased cell growth, and miR-376c-3p also favored cell migration of SK-Mel-28 and Ma-Mel-19 in the scratch test closure assay. These data suggest that prometastatic activity conferred by the P2X7R is, at least in part, dependent on the vesicular release of miRNAs. Previous data on the role of miR-495-3p, miR-376c-3p, and miR-6730-3p in cancer is limited. A few studies reported the association of miR-376c-3p with cancer promotion [37–39]. All three miRNAs are predicted to affect several biochemical pathways, including neuroactive ligand receptors as P2Xs, and molecules associated with cancer transformation or metastatic spreading, such as the PI3K/Akt and mTOR axis or tight junctions and cell adhesion molecules. The ability of P2X7R antagonist A740003 to reduce vesicle release of miR-495-3p, miR-376c-3p, and miR-6730-3p and tumor growth and dissemination strongly suggests that P2X7R-targeting might offer a new therapeutic opportunity to interfere with the pro-metastatic and niche-preconditioning activity of EV [21]. Our data show that the P2X7R is overexpressed and associates with a poor prognosis in metastatic melanomas. Tumor and metastases-promoting activity is also due to P2X7R ability to drive the release of EVs containing growth and migration-promoting miRNAs.

## Materials and methods

### P2X7R expression analysis

Publicly available RNA sequencing data for P2X7R were downloaded from the Cancer Genome Atlas database (TCGA) through the Firebrowse repository (http://firebrowse.org/; release 01/28/2016) in all tumor types and skin cell melanoma (SKCM) cohort. Association of P2X7R expression with overall survival was assessed using Kaplan–Meier curve and long rank test after applying a threshold of 10.35 to RSEM log2 expression data using GraphPad Prism Software (GraphPad, La Jolla, USA).

### Patients samples and ethics

Patient specimens analyzed by immunohistochemistry were part of a Tissue Array Slide (CK2 Human, malignant melanoma, Super Bio Chips, Korea). We selected 25 samples from primary tumors and 14 from metastatic formations. cDNA samples analyzed by RT-PCR were part of TissueScan Melanoma Tissue qPCR Panel I MERT01 and MERT02 (OriGene). cDNA melanoma samples were subdivided according to the diagnostic phase and site of metastasis, reported by manufacturers and checked for accuracy by Dr. Dika, in four groups: stage III, stage IV with skin metastasis, stage IV with lung metastasis and stage IV with metastasis in other distant organs including brain and liver. Ethics approval for human samples was obtained by the vendors; our study was performed in accordance with the declaration of Helsinki.

### Immunohistochemistry

Tissue array slides were stained as described in ref. [13] using an anti-P2X7R antibody (P8232 clone, Sigma-Aldrich) at 20 μg/mL and hematoxylin as the counterstain. Images were captured with a Nikon Eclipse H550L microscope using the NIS-Element software (Nikon). Each sample was attributed a high or low positivity score by three independent operators.

### Real-time quantitative RT-PCR for P2X7R A and B

Amplification was performed with TaqMan probes for P2X7RA, P2X7RB, and GAPDH as reference mRNA. Pre-designed and custom primers were those described in ref. [14]. A comparative CT experiment (ΔΔCT) was run to determine the fold increase of the target cDNA relative to the Te85 cell line reference sample [13].

### Cell cultures and transfections

Human Ma-Mel-19 (ECACC 13012460) and Sk-Mel-28 (ATCC HTB-72), and Mouse B16-F10 (ATCC CRL-6475) melanoma cell lines were purchased from Sigma Aldrich and periodically tested with MycoAlert^TM^ kit (Lonza, Switzerland). Cells were cultured in RPMI-1640 medium supplemented with 10% FBS, 100 U/mL penicillin, and 100 mg/mL streptomycin (Euroclone). Sk-Mel-28 luciferase Luc2 (Promega, Italy) stable transfection was obtained with Lipofectamine LTX (Thermo Fisher) and by selection with hygromycin B (0.2 mg/mL; Sigma-Aldrich). miR-495-3p, miR-6730-3p, miR-376c-3p, or scramble control (mirVana miRNA mimics, Ambion) were transfected with Lipofectamine RNAimax (Thermo Fisher).

### P2X7R activity assays

P2X7R activity as Ca^2+^ and Ethidium permeable pore was tested as previously described in ref. [40].

### Cell counts

30 × 10^3^ Ma-Mel-19 or Sk-Mel-28 cells were cultured in RPMI-1640 medium and counted as described in ref. [14]. Whenever required, A740003 and AZ10606120 (Tocris Bioscience, UK) were added, respectively, at 20 or 2 μM.

### Soft agar assay

The soft agar colony formation assay was performed as described in ref. [41]. Briefly, 5 × 10^4^ cells per well were seeded in an RPMI 0.8% agarose gel with either 20 μM A740003, 2 μM AZ10606120, or PBS and stratified on an RPMI 0.6% agarose gel.

### Scratch recovery assay

The scratch recovery assay was performed as previously described in refs. [42, 43]. The scratch recovery rate on time 0 was calculated with ImageJ software.

### Murine models

1 × 10^6^ Sk-Mel-28 Luc2 cells were injected into the tail vein of the 6-week-old female athymic nude-Foxn1nu mice (Envigo, Italy). The animals were randomized, and the operator was blinded to the group of allocation. Mice were intra-peritoneum (i.p.) injected with P2X7 antagonist A740003 (50 mg/kg) or vehicle (PBS, 0.005% DMSO) every third day from the inoculum. Cell body dissemination was evaluated thanks to Luc2 luciferase photon emission with an IVIS Lumina Luminometer (Perkin Elmer, USA). Mice were i.p. injected with 150 mg/kg d-luciferin (Promega), and luminescence was captured from ventral view every third day for a total of 33. Photon emission was quantified using the Living Image^®^ software (Perkin Elmer). 2 × 10^5^ B16-F10 cells were injected into the tail vein of C57bl/6 5–6 weeks old female mice (Envigo). P2X7 antagonist A740003 (50 mg/kg) or vehicle (PBS, 0.005% DMSO) were administered i.p. every third day. On day 18, following sacrifice, mice lungs were explanted, and the number of visible metastasis was evaluated. All animal procedures were approved by the University of Ferrara Ethics committee and the Italian Ministry of Health (Italian D.Lgs 26/204).

### Microscopy

Confocal experiments were performed in the following solution: 300 mM sucrose, 1 mM K_2_HPO_4_, 1 mM MgSO_4_, 5.5 mM d-glucose, 20 mM Hepes, 1 mM CaCl_2_, pH 7.4. Cell membranes were labeled with 1 µM PKH26GL (Sigma), while ATP and nucleic acids were stained 1 µM Quinacrine (Sigma). Images were acquired at 30 s intervals for 15–30 min with a Zeiss LSM 510 microscope after stimulation with 500 µM BzATP. For electron microscopy, cells were incubated for 10 min with or without 3 mM ATP, detached from the flasks with 2 mM EDTA cold PBS, centrifugated at 1100 rpm, fixed with 2.5% glutaraldehyde and processed by the Electron Microscopy Center of the University of Ferrara.

### Ultracentrifugation and nanoparticle tracking analysis

Confluent cells were incubated in saline solution (see above) with or without 3 mM ATP for 30 min at 37 °C. Cells and debris were removed by centrifugation at 2000 × *g* for 20 min at 4 °C. MV fraction was pelleted at 18,000 × *g* for 40 min at 4 °C. The remaining supernatant was centrifuged at 100,000 × *g* for 4 h at 4 °C to obtain EXO. For nanoparticle tracking analysis, MV and EXO fractions were resuspended in 60 μl of filtered PBS and diluted 1:100–1:500 in particles-free PBS. EV were tracked using the Nanosight system (Nanosight LM10, Malvern, UK), according to the manufacturers. Data were analyzed with NanoSight Software NTA 3.2 Dev Build 3.2.16.

### Characterization of EV by immunoblotting

MV and EXO lysates were prepared in RIPA buffer with Halt^TM^ Protease and Phosphatase inhibitor cocktail, EDTA-free 100× (Thermo Fisher). MV and EXO lysates were loaded in 4–12% NuPAGE Bis–Tris precast gels (Thermo Fisher), proteins were separated and transferred onto a nitrocellulose membrane (Amersham Protran, USA). Membranes were incubated overnight at 4 °C with the following antibodies: anti-P2X7 (1:300, P8232, Sigma-Aldrich), anti-calnexin (1:5000, GTX109669, GeneTex), anti-Alix (3A9) and anti-flotillin-1 (1:1000, Exosomal Marker Antibody Sampler Kit, Cell Signaling Technology). Secondary anti-rabbit (1706515, BIORAD) or anti-mouse (1706516, BIORAD) antibodies were applied at a 1:3000 dilution.

### NGS sequencing and miRNA validation by RT-PCR

RNA was extracted from EV with QIazol Lysis Reagent and miRNasy^®^Mini Kit (Qiagen), and its sequencing was performed with QIAseq miRNA library kit (Qiagen) and NextSeq500 sequencer (Illumina). Raw data were proceeded by Qiagen GeneGlobe Data Analysis Center to obtain miRNA quantification, with Unique Molecular Indices (UMIs) correction for potential PCR and sequencing bias. DESEQ2 [44] normalized data were imported in GeneSpring software (Agilent Technologies) for data analysis. A 1.5-fold change filter and *p* < 0.05 at moderated *t*-test for group comparison were used. NGS raw data are available through European Nucleotide Archive (http://www.ebi.ac.uk/) under accession number PRJEB45302. To validate the overexpression of miR-495-3p, miR-376c-3p, and miR-6730-3p in vesicular RNA, cDNA templates were prepared using the TaqMan^®^Advanced miRNA cDNA Synthesis kit (A28007, Thermo Fisher). The analysis of miRNAs expression levels was performed using TaqMan^®^Advanced miRNA Assays using RNU6-1 as endogenous control (Thermo Fisher).

### miRNAs target prediction

DIANA-miRPath 3.0 web-server was used to predict interactions between miR-495-3p, miR-376c-3p, miR-6730-3p, and predicted target mRNAs using TargetScan predictive tool and Kyoto Encyclopedia of Genes and Genomes (KEGG) as a reference set for the pathway analysis and the *p*-value of enrichment was calculated.

### Statistics and data availability

Fisher’s exact test was used to compare data obtained by immunohistochemistry and the incidence of metastasis formation in C57bl/6 melanoma-bearing mice. Other data are shown as mean ± standard error of the mean (SEM). Significance was calculated assuming equal standard deviations and variance with a two-tailed Student’s *t*-test. All tests were performed with the GraphPad Prism Software. For in vivo experiments, a sample size of eight animals per condition was computed a priori with the G*power software [45] based on previous data obtained with in vivo administration of A740003 [9] and assuming an effect size of 2 and a power of 95%. Full data sets will be made available upon reasonable request to the corresponding author.

## Supplementary information

Movie 1

Movie 2

Movie 3

Supplementary figure 1

Supplementary figure 2

NPJ check list
