# Supplementary figures and images for: P2X7 promotes metastatic spreading and triggers release of miRNA-containing exosomes and microvesicles from melanoma cells
Source: Cell Death Dis. 2021 Nov 16;12(12):1088. doi: 10.1038/s41419-021-04378-0 (PMC8599616; doi:10.1038/s41419-021-04378-0)

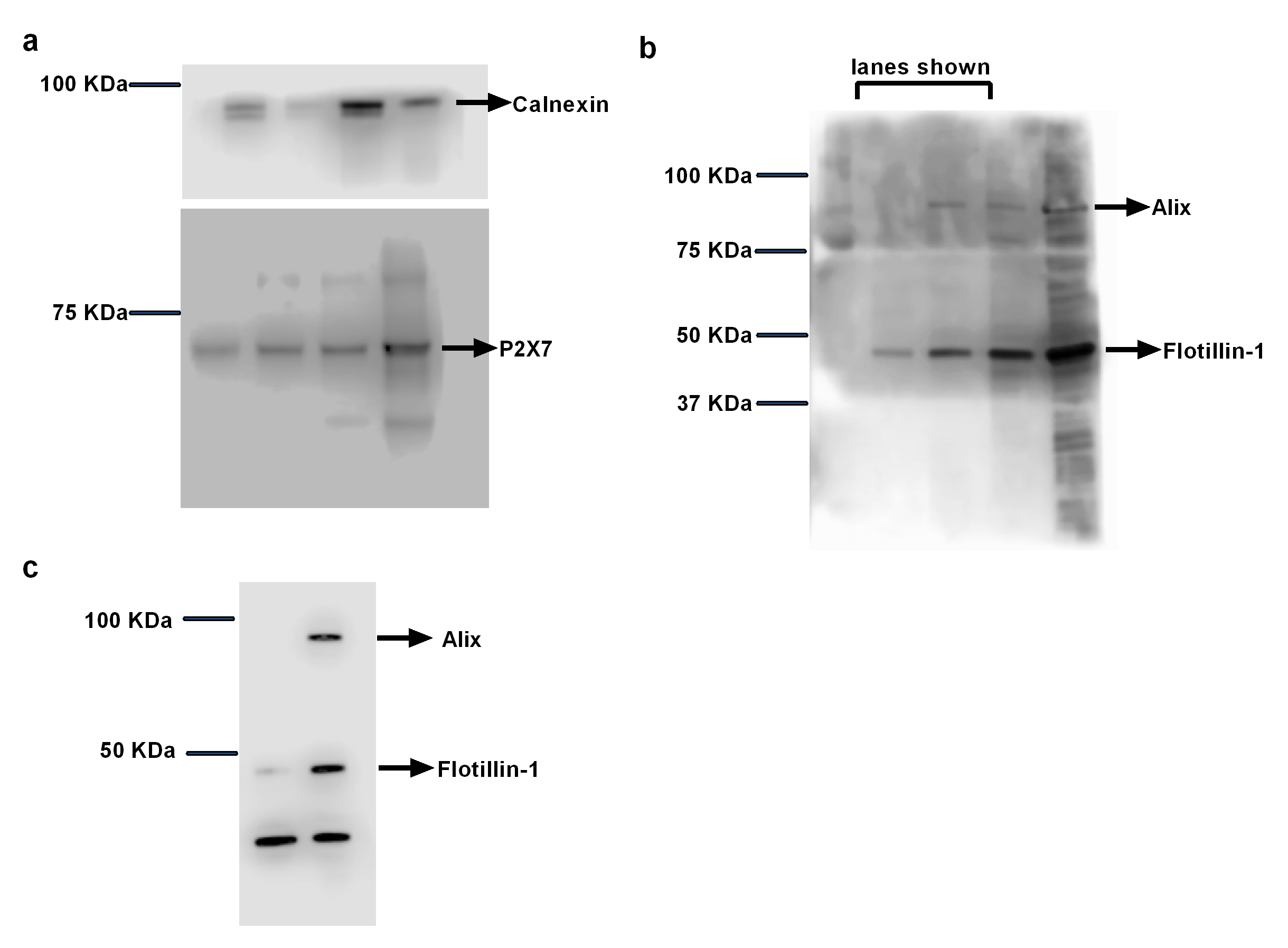

Supplement: Supplementary file 4 — Supplementary figure 1 [file 41419_2021_4378_MOESM4_ESM.jpg]

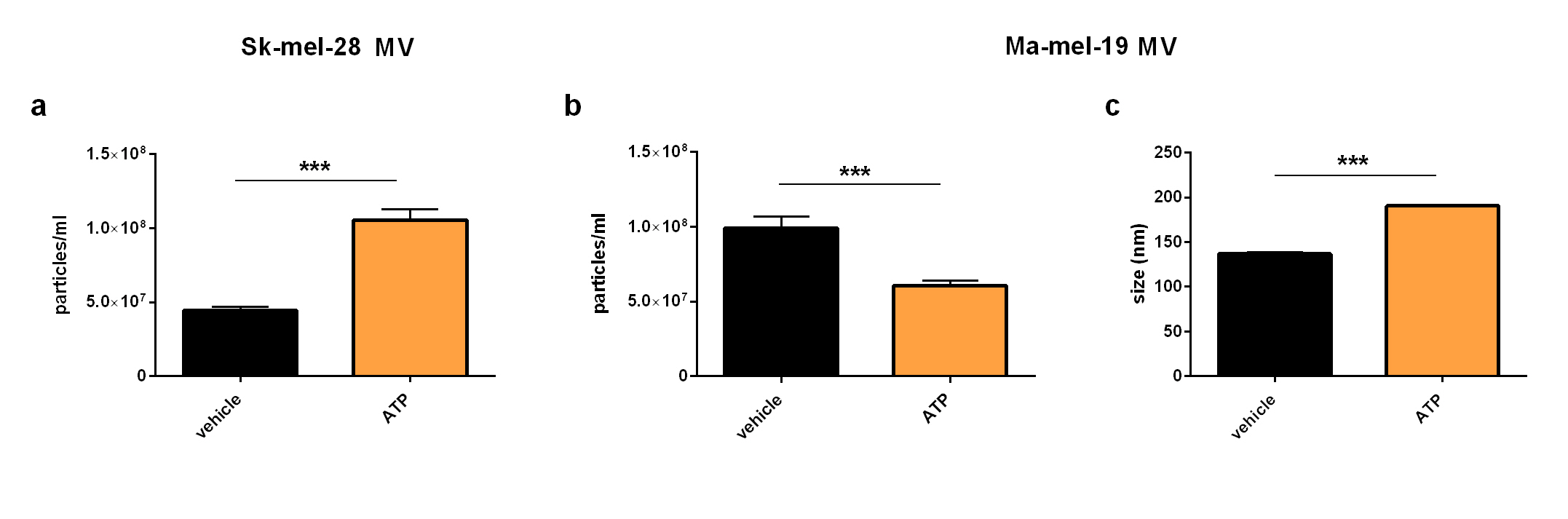

Supplement: Supplementary file 5 — Supplementary figure 2 [file 41419_2021_4378_MOESM5_ESM.jpg]
